# In Vivo Effect of Resveratrol-Cellulose Aerogel Drug Delivery System to Relieve Inflammation on Sports Osteoarthritis

**DOI:** 10.3390/gels8090544

**Published:** 2022-08-29

**Authors:** Ningxin Cui, Zhen Xu, Xinyu Zhao, Meng Yuan, Leiyu Pan, Tianfeng Lu, Ai Du, Lili Qin

**Affiliations:** 1 Sports and Health Research Center, Department of Physical Education, Tongji University, Shanghai 200092, China; 2 School of Physics Science and Engineering, Tongji University, Shanghai 200092, China

**Keywords:** osteoarthritis, cellulose aerogel, resveratrol, anti-inflammatory

## Abstract

Resveratrol (Res) is a plant extract with strong anti-inflammatory, antioxidant and anti-aging biological activities. However, Res is limited by its disadvantages, such as poor solubility, rapid metabolism and low bioavailability. In this study, the Resveratrol-loaded TEMPO-oxidized cellulose aerogel (RLTA) drug delivery system was prepared by the method of “dissolution-freeze-drying” and characterized by a series of analysis. Then the blood biochemical indexes and HE staining were measured and analyzed in animal experiments. The in vivo results showed that RLTA can decrease the levels of TNF-α and IL-6 inflammatory factors in the synovial fluid. Furthermore, the molecular mechanism was investigated through the analysis of silent information regulator 2 homolog 1 (Sirt1) protein expression, which suggested that RLTA could upregulate the expression of Sirt1 and mediate the P38 signaling pathway, thereby inhibiting the expression of COX-2 and MMP13 which can suppress the levels of IL-6 and TNF-α inflammatory factor. These results reveal that cellulose aerogel is a promising candidate for drug delivery and RLTA has great potential application for the treatment of sports osteoarthritis.

## 1. Introduction

Osteoarthritis (OA) is characterized by degeneration of articular cartilage, inflammation of the synovium and changes in subchondral bone leading to joint abnormalities, instability, pain and loss of function. At the molecular level, it is characterized by imbalance between chondrocyte anabolism and catabolism [1]. Sports osteoarthritis is a joint lesion caused by excessive wear and joint trauma, which can lead to joint degeneration, joint pain, inflammatory changes and so on. The main pathogenic factor is abnormal joint load distribution, such as repeated shocks, torsional loads, and sudden increased stress applied to joint surfaces during exercise. At present, drug therapy, physical therapy, manipulation therapy, exercise therapy, arthroscopy and operations are considered as common treatments of knee osteoarthritis [2].

Resveratrol (Res) is a natural substance extracted from plants. Recently, it has attracted more attention due to its various pharmaceutical characteristics, such as anti-inflammatory, antioxidant, anti-aging, anti-tumor and cardiovascular protection effects [3]. Res has non-steroidal anti-inflammatory activity and can be applied to the treatment of inflammatory diseases. It has been found to exert anti-inflammatory effects by dephosphorylating JNK and P-IKK β inflammatory signaling pathways, inhibiting the activation of NF-κB and P38 MAPK pathways, and inhibiting the activation of NLRP3 inflammasome, thereby inhibiting the synthesis of cellular inflammatory cytokines such as IL-1 and IL-6 and proinflammatory mediators [4,5,6,7]. However, Resveratrol has some unfavorable properties; it can be rapidly absorbed, metabolized and excreted by organisms, indicating that Res has poor bioavailability [8,9]. Therefore, in order to improve the solubility and stability of Res, the development of new drug carriers has become the focus of research in recent years.

It has been found that porous material used as a drug carrier can effectively reduce drug particle size, facilitate drug absorption, and avoid rapid decomposition of drugs in gastrointestinal tract. Aerogel, as a typical porous material, has the characteristics of extremely low density, low thermal conductivity, high specific surface area and high porosity [10,11,12,13]. In recent years, scientists have turned their attention to the development of natural polymers because of the biodegradability of these materials [14,15]. Cellulose, as a kind of biomass material, has good biocompatibility and high safety. Cellulose aerogel as a drug carrier has been widely used in the field of medicine. Some studies examined tempo-oxidized nanocellulose loaded with gentamicin, and the drug was basically released within 420 min. A bacterial cellulose membrane loaded with flavonoids (quercetin, morin and baicalein) had a good antibacterial effect on Escherichia coli and Staphylococcus aureus. Moreover, the fixation of hydrophobic protein coated nanoparticles in nanocellulose matrix, which reduced the particle size of the drug itraconazole, increased its stability and resulted in a good sustained-release effect [16]. Therefore, cellulose aerogel can be used as a drug carrier and has great development potential in the field of biomedicine.

**Scheme 1:**
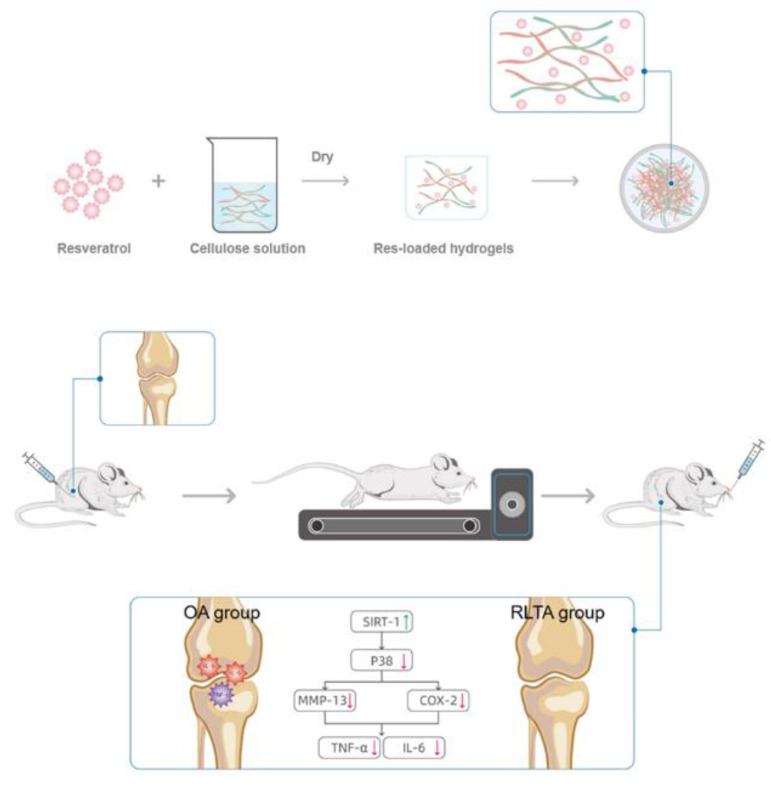
Schematic diagram of the synthesis process of RLTA and the regulatory mechanism of RLTA in the treatment of sports osteoarthritis.

In the previous study, the Resveratrol-loaded TEMPO-oxidized cellulose aerogel (RLTA) was successfully prepared and characterized [17]. In this study, RLTA was designed as an anti-inflammation drug against sports osteoarthritis. RLTA could effectively reduce the particle size of resveratrol, which would be conducive to drug absorption and sustained release. The in vivo animal experiments showed that RLTA could alleviate the progression of OA through activating Sirt1 and inhibiting P38 mediated inflammatory pathway (Scheme 1). In summary, the cellulose aerogel exhibited a promising drug delivery platform and RLTA has a good potential for application in the treatment of sports osteoarthritis.

## 2. Results and Discussion

### 2.1. Characterization of Materials

**Figure 1:**
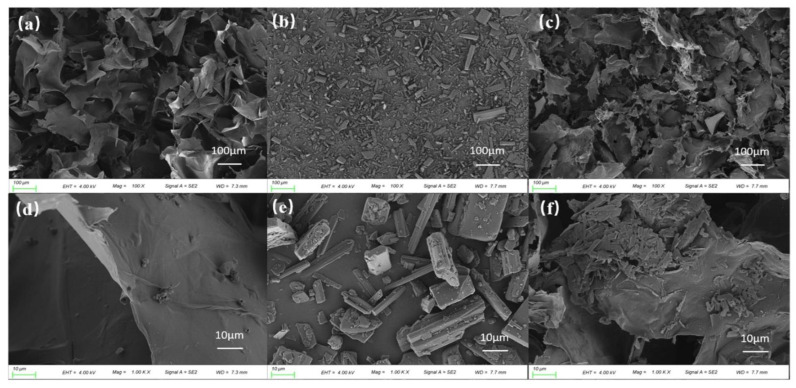
The SEM images of materials and drugs: (**a**,**d**) The SEM images of TC aerogel. (**b**,**e**) The SEM images of Res. (**c**,**f**) The SEM images of RLTA.

**Figure 2:**
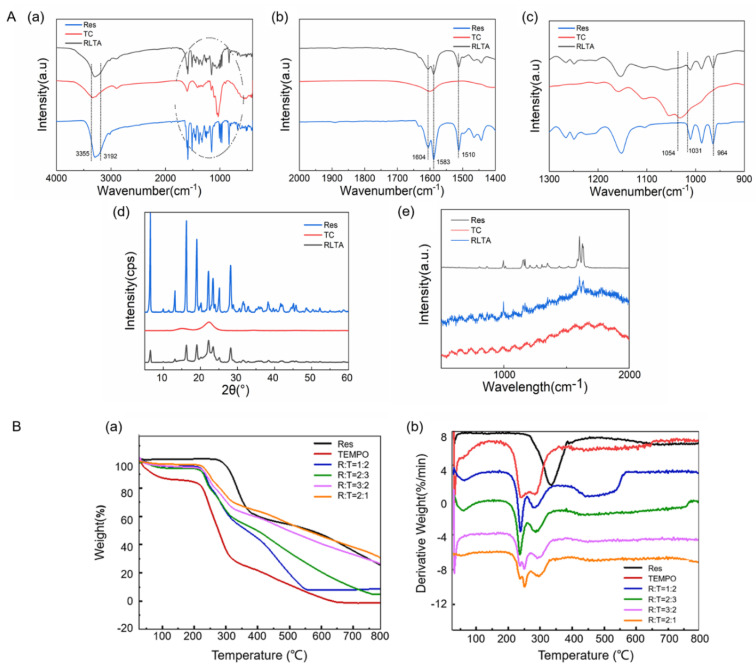
(**A**) Characterization of materials and drugs: (**a**–**c**) FTIR of Res, TC aerogel and RLTA. (**d**) XRD of Res, TC aerogel and RLTA. (**e**) Raman of Res, TC aerogel and RLTA; (**B**) Thermogravimetric curves of TC aerogel, Res and RLTAs. (**a**) TG of Res, TC aerogel and RLTA; (**b**) DTG of Res, TC aerogel and RLTA.

As shown in Figure 1a, the skeleton of pure Tempo-oxidized cellulose (TC) aerogel was flaky and fibrous and fibres were intertwined with each other. Pure Res comprised clustered rod-like structures (Figure 1b). The structure of RLTA was long fibrous and rod-like, which was similar to that of TC aerogel, and Res adhered to the TC surface and filled the pores between the skeletons (Figure 1c). This phenomenon was more obvious in higher resolution shots, as shown in Figure 1d–f. Figure 2A shows the full spectra of TC aerogel, Res and RTLA. The relatively weak peaks at 1054 cm^−1^ [18] and 1031 cm^−1^ can be attributed to C–O [19,20,21] stretching vibration, and the characteristic peaks related to Res were 1604 cm^−1^, 1583 cm^−1^, 1510 cm^−1^ and 964 cm^−1^ [22,23], respectively. In Figure 2A(c), RLTA shows the same C–O peak position as TC aerogel at 1054 cm^−1^ and 1031 cm^−1^. In Figure 2A(b,c), according to the spectra of Res and RLTA, the characteristic peaks of C=C tensile vibration at 964 cm^−1^ and benzene ring vibration at 1604 cm^−1^, 1583 cm^−1^ and 1510 cm^−1^ are evident. RLTA shows characteristic peaks of TC aerogel and Res, indicating the successful recombination of Res and TC aerogel [24,25] The crystal types of TC, Res and RLTA were confirmed by X-ray diffraction (Figure 2A(d)). TC aerogel was a disordered nanocrystalline material with crystal peaks similar to parent cellulose [26,27]. When TC and Res were recombined, there were still five obvious diffraction peaks of Res crystal. As can be seen from Figure 2A(d), the crystal form was almost unchanged after recombination [28,29]. In addition, X-ray diffraction analysis results showed that the grain size of RLTA decreased greatly after recombination. Figure 2A(e) shows the Raman spectra of three samples. Res is characterized by two characteristic peaks at 1123 cm^−1^ and 1656 cm^−1^. Raman spectroscopy showed that the spectrum of RLTA contained all characteristic peaks of resveratrol and TC aerogel, indicating the presence of resveratrol in RLTA [30]. Compared with pure drugs, all peaks were at the same location. These results indicated that the drug retained its intact structure during preparation.

As shown in Figure 2B, the thermal decomposition of the samples was analyzed by thermogravimetric test. At 25–230 °C, the TEMPO oxidized cellulose aerogel first showed a small amount of weight loss, which may be caused by a small amount of water remaining during freeze-drying or the evaporation of a small amount of water absorbed by the sample; the rapid weight loss at 220–300 °C was about 60%, the initial decomposition temperature was 220 °C, and then there was a slow weight loss. Res showed rapid weight loss at 300–360 °C; the initial decomposition temperature was 300 °C, and then the weight loss was slow. The initial decomposition temperature of drug-loaded cellulose aerogel is lower than that of pure resveratrol drug and higher than that of TEMPO-oxidized cellulose aerogel, which is closer to the initial decomposition temperature of TEMPO-oxidized cellulose. Rapid weight loss occurs at 250–350 °C, followed by slower weight loss. After 710 °C, the weight loss of the resveratrol of composite cellulose was smaller than that of pure resveratrol drug. It is speculated that the resveratrol after loading has higher thermal stability. When the ratio of Res and TC aerogels is different, the drug loading rate is slightly different. According to Formula (1), the loading rate of RLTA can be calculated as 18%.

### 2.2. The Release Behavior of RLTA

**Figure 3:**
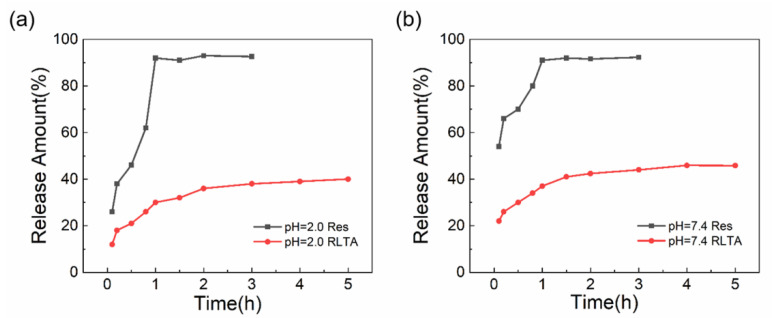
Simulated release diagrams of Res and RLTA under different conditions: (**a**) artificial gastric juice pH = 2.0. (**b**) phosphate buffer pH = 7.4.

Res and RLTA were placed in simulated gastric fluid (pH = 2.0) and phosphate buffer solution (pH = 7.4) to conduct release behavior studies, and the stability of RLTA after recombination was also examined. Figure 3 shows the change of the release amount of Res at different times. It was found that sudden drug release occurred in the first 15 min, which was analyzed to be the drug desorption process adsorbed on the surface of cellulose aerogel. After that, the release rate continued slowly and stopped after 5 h. The drug release in artificial gastric solution was 35.6%, and that in phosphate buffer was 45.9%, while the release of Res in the same amount reached 91% at 5 h. In vitro drug release studies showed that Res could be released more stably and continuously when Res and TC aerogel were successfully combined. The release rate of the drug is related to the matrix material. Cellulose aerogel as a matrix could enable the drug to possess the sustained release profile [31]. In our previous study, we found that Res fitted a first-order release model and the release rate was related to the concentration difference [17]. Therefore, the in vivo effect of RLTA was designed in the following study.

### 2.3. Biosafety of RLTA

#### 2.3.1. Changes in Body Weight of Rats

Changes in body weight may reflect the progression of arthritis and general health status, while weight loss higher than that in the arthritic group may indicate toxicity. The effect of drug administration on the body weight of rats was studied. It was found that the body weight of rats in the five groups showed an overall upward trend during 3 weeks of treatment. After the modeling, the body weight of the blank group (Control) was at the highest state without inflammation and running platform modeling, and then maintained a slow increase. Although the body weight of model group (OA) also increased slowly, it was in the lowest state compared with other groups, which was speculated to be due to the loss of appetite and the decline of body function caused by inflammation, resulting in the overall low body weight. The body weight associated with the drug loading system (C+R) and pure drug group (Res) was lower, which was speculated to be due to the fact that Res itself had the function of improving glucose metabolism, resulting in the effect of weight loss [32]. However, the body weight of the C+R group was lower than that of the Res group, which was speculated to be due to the slow release of the drug loading system and the shrinkage of particle size, which was more conducive to the absorption of the drug, and the weight loss effect was more obvious. There was significant difference between blank group (Control) and other 4 groups (*p* < 0.05), but no significant difference was found between the other 4 groups.

#### 2.3.2. Blood Biochemical Indices of Rats

**Figure 4:**
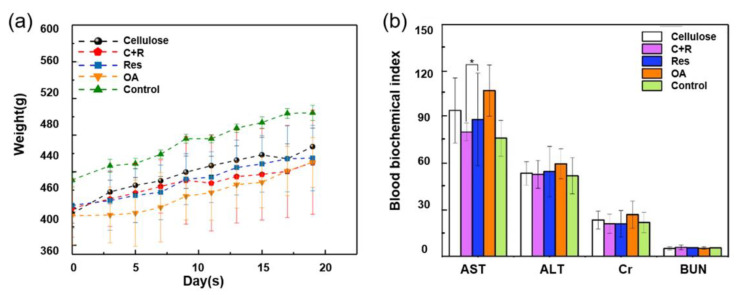
(**a**) The weight of the rats in each group during the whole experimental period. (**b**) Effect of administration on liver function (AST, ALT) and renal function (Cr, BUN) of rats. (* *p* < 0.05).

The effects of drug administration on liver function and renal function of rats were evaluated by analyzing the indexes of aspartate aminotransferase, glutamic-pyruvate transaminase, creatinine and urea nitrogen [33]. As shown in Table 1, only AST showed significant differences between the intervention group and the model group (*p* < 0.05), while the other three groups showed no significant differences. In the evaluation indexes of liver function (AST and ALT), it can be seen that the RLTA group had a better effect on improving liver function. In the evaluation indexes of renal function (Cr and BUN), Cr level was lower than that of the model group, and BUN level was similar in each group. According to the data in Table 1 and Figure 4b, after RLTA was administered to rats, there was no damage to important organs, which proved that RLTA had good biosafety.

### 2.4. In Vivo Therapeutic Effect of RLTA in Osteoarthritis

#### 2.4.1. Micro-CT and HE

**Figure 5:**
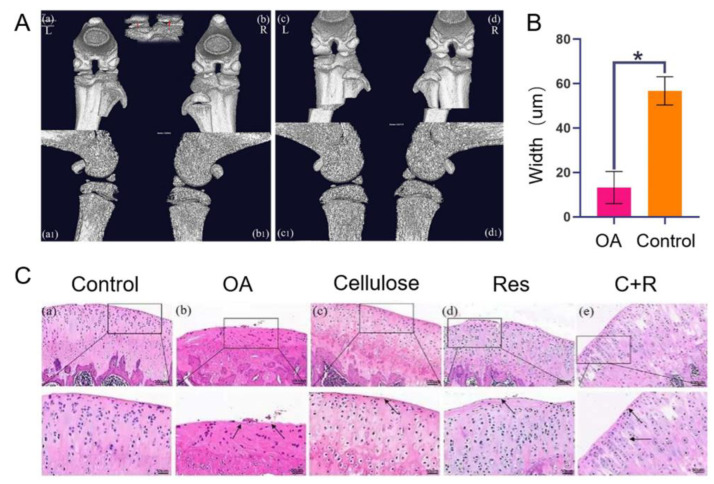
(**A**) Micro-CT examination of knee joint: (**a**,**b**) Positive bitmap of inflammation group; (**a1**,**b1**) Lateral map of inflammation group; (**c**,**d**) orthographic map of control group; (**c1**,**d1**) Lateral map of the control group; (**B**) Knee spacing measurement (* *p* < 0.05); (**C**) H&E staining images of the weight-bearing area on the medial edge of tibial end of knee cartilage in each group: (**a**) Control: normal Control group; (**b**) OA: arthritis model group; (**c**) Cellulose: the treatment group of Cellulose; (**d**) Res: Res treatment group; (**e**) C+R: Cellulose aerogel loaded with Res treatment group (N = 5).

Micro-CT showed that the left and right knee spaces in the control group were the same [34,35], while the right knee space in the inflammation group was significantly narrowed (Figure 5A). The vertical distance between the medial lateral condyle of femur and tibial plateau was measured, with the lateral condyle as the standard. Statistical analysis showed that there was significant difference in the right knee space between the control group and the inflammation group (Figure 5B). Micro-CT results showed that the rat model of knee arthritis was successfully constructed.

HE staining results showed that compared with the normal group, the surface cartilage of the model group was damaged, and the number of chondrocytes was seriously reduced and disordered. The surface cartilage of the cellulose group, Res group and the C+R group were smoother than that of the OA group, close to the Control group, and the number of chondrocytes also increased significantly. The cell stratification of the C+R group was clearer than that of the Res group and cellulose group (Figure 5C).

#### 2.4.2. Influence of IL-6 and TNF-α in Articular Fluid

**Figure 6:**
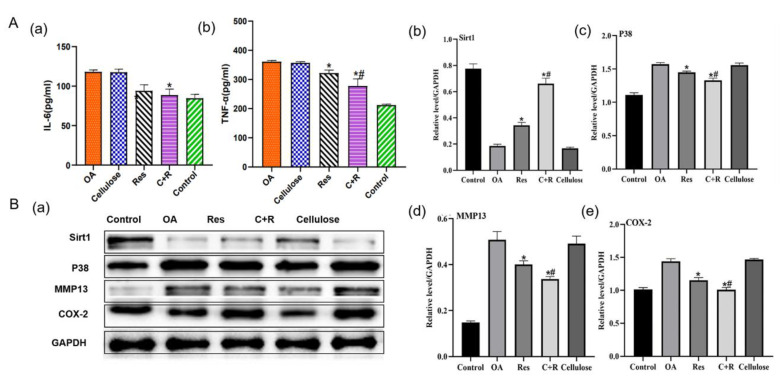
(**A**) Expression of IL-6 (**a**), TNF-α (**b**) in articular fluid of rats (* *p* < 0.05; *# *p* < 0.05); (**B**) Expression of Sirt1 (**b**), P38 (**c**), MMP13 (**d**) and COX-2 (**e**) proteins in rat articular cartilage: (**a**) Western blot analysis of several proteins. (**b**–**e**) Average gray relative value of each protein with GAPDH as internal reference. (* *p* < 0.05; *# *p* < 0.05).

Arthritis is caused by the release and persistence of many pro-inflammatory cytokines [36,37]. TNF-α and IL-6 are representative inflammatory markers and are closely related to synovial inflammation and degradation of articular cartilage matrix. Studies have shown that the expression of TNF-α is positively correlated with knee osteoarthritis inflammation. Other studies have found that the expression of IL-6 is closely correlated with the prevalence and incidence of knee osteoarthritis, and can be used as an indicator to predict its severity [38]. The results showed that after 3 weeks of treatment, the expressions of IL-6 and TNF-a in the articular fluid of the pure drug group (Res) and the drug loading system group (C+R) decreased compared with the model group (OA), with significant difference compared with the OA group (*p* < 0.05), and the expression of the C+R group was significantly lower (Figure 6A). To sum up, RLTA has a better therapeutic effect on knee osteoarthritis. It is speculated that the particle size of Res decreases after it is combined with TC aerogel, which is conducive to drug absorption and has a better treatment effect on inflammation.

#### 2.4.3. Influence of Articular Cartilage Related Protein Expression

At present, more and more studies have shown that the decrease of Sirt1 level is closely related to many inflammatory diseases, and Sirt1 is considered to be a negative regulator of inflammatory response in vivo [39]. Western blot results showed that Sirt1 had the lowest expression in the OA group and the highest expression in the RLTA group (Figure 6B(b)). The expression of P38, Matrix metalloproteinase13 (MMP13) and Cyclooxygenase 2 (COX-2) protein showed an opposite trend to that of Sirt1, with the highest level in the OA group and the lowest level in the RLTA group (Figure 6B). Sirt1 can be used for anti-inflammatory treatment by inhibiting the phosphorylation of P38. P38 is an essential enzyme for activating MMP13. In the development of arthritis, the P38 signaling pathway regulates the expression of inflammatory factors, participates in the synthesis of COX-2 and induces the expression of MMP13 which can destroy articular cartilage [40]. The occurrence of arthritis is caused by the release and persistence of many pro-inflammatory cytokines such as TNF-α and IL-6, which are representative inflammatory markers. Therefore, Sirt1 acting as a negative regulator of inflammatory responses can affect the expression of pro-inflammatory cytokines such as TNF-α and IL-6. The results of this experiment showed that the Sirt1 protein expression level was higher than that of the OA group after administration of the intervention, and the Sirt1 protein level in the RLTA group was higher than that in the Res group, indicating that Res has the effect of activating the Sirt1 protein. Moreover, RLTA may have a better therapeutic effect due to the reduction of drug particle size and its slow-release effect. The protein levels of P65, P38 and MMP13 showed an opposite trend to Sirt1, indicating that Sirt1 played a certain inhibitory effect on several proteins, and the inhibitory effect was more obvious in the RLTA group due to the high Sirt1 level. The mechanism of Sirt1’s involvement in anti-inflammatory regulation in osteoarthritis is still uncertain. Currently, it is believed that Sirt1 plays a role mainly through inhibition of the NF-κB signaling pathway or regulation of the P38 MAPK pathway. It has suggested that Sirt1 has an anti-inflammatory function by interfering with the NF-κB pathway and interacts with the P65 subunit of NF-κB. In macrophages and endothelial cells, Sirt1 deacetylates the P65 subunit of NF-κB, thereby down-regulating the expression of various pro-inflammatory cytokines [41]. Some studies have also shown that Sirt1 can also provide anti-inflammatory treatment by inhibiting the phosphorylation of P38 [42]. In order to explore the therapeutic effect of RLTA on knee osteoarthritis induced by exercise, the expression levels of Sirt1, NF-κB, P38, COX-2 and MMP13 in chondrocytes were detected. It was found that the expression level of Sirt1 protein in the RLTA administration group was higher than that in the OA group, and the Sirt1 protein level in the RLTA administration group was higher than that in the Res group, while the protein levels of P38, COX-2 and MMP13 showed an opposite trend to Sirt1. It is speculated that Sirt1 has a certain inhibitory effect on both signaling pathways, and the RLTA group has a more obvious inhibitory effect on P38, COX-2 and MMP13 due to the high level of Sirt1. By investigating the P38 signaling pathway and downstream inflammatory signaling factors, it was speculated that the drug inhibited the expression of COX-2 and MMP13, and down-regulated the levels of IL-6 and TNF-α inflammatory factors by activating the P38 signaling pathway.

## 3. Conclusions

This study focused on the therapeutic effect of RLTA on sports osteoarthritis. First, RLTA was prepared by the “dissolution-lyophilization” method. Then, the characterization of Res, TC aerogel and RLTA was tested and analyzed. It was found that cellulose aerogel as a drug carrier can effectively reduce the particle size of the drug and improve the drug activity which can enhance the sustained release effect. Furthermore, a rat model of sports osteoarthritis was constructed. Through the analysis of rat body weight, biochemical indexes and histopathological manifestations, it was found that the drug delivery system had a good therapeutic effect on OA. Finally, the anti-inflammatory mechanism suggested that RLTA could activate Sirt1 expression and downregulate the P38 signaling pathway. It can thereby inhibit the expression of COX-2 and MMP13 which could suppress the levels of IL-6 and TNF-α inflammatory factors. In conclusion, cellulose aerogel could serve as a novel drug carrier and RLTA had potential application value in the treatment of sports osteoarthritis.

## 4. Materials and Methods

### 4.1. Materials

Tempo Oxidized Cellulose was purchased from Ningbo Solectron Nanotechnology Co., Ltd. (Shanghai, China); Resveratrol (purity 99%) was purchased from Aladdin Reagent Shanghai Co., Ltd. (Shanghai, China); Deionized water was purchased from School of Environment and Engineering (Tongji University, Shanghai, China).

### 4.2. Preparation of RLTA

The detailed preparation process and characterization of RLTA were described in our previous study [17]. TC aerogel was prepared by dissolution-freeze-drying method. 135 mg cellulose and 16.75 mL water were placed in a glass bottle and stirred at 500 RPM for 20 min. After dissolution, it was quickly frozen with liquid nitrogen and placed in a freeze dryer for freeze drying. 90 mg Res was mixed with 2 mL alcohol and stirred until fully dissolved. Then, 132 mg cellulose was mixed with 16.75 mL water and stirred for 20 min (500 rpm). Next, the two dissolved solutions were mixed and stirred under dark for 10 min. After mixing evenly, the mixed solution was quickly frozen with liquid nitrogen. Finally, the sample was dried in a freeze dryer for 24 h to obtain RLTA.

### 4.3. Characterizations

Scanning electron microscopy (SEM) was used for analyzing the Surface Morphology of TC aerogel, Res and RLTA. The Philips-XL30FEG electron microscope of Thermo Fisher Technology Co., LTD was used for SEM. TC aerogel, Res and RLTA were sprayed with gold and observed under a microscope. The surface functional groups were measured by Fourier transform infrared (FTIR) using a Thermo Fisher Nicolet 6700 infrared spectrometer. The sample powder was dispersed into KBr matrix at a ratio of 1:100 and pressed, and detected in the wave number range of 900–4000 cm^−1^. X-ray diffraction used the X-ray powder diffractometer from Bruker, Germany. The samples were tested at a voltage of 20 KV, a current of 50 mA, and a sweep range of 5 to 60° at 2θ. Raman spectroscopy was used to characterize the structural electronic properties, crystal structure and disorder of Res, TC aerogel and RTLA, and the induced structural changes during the preparation were measured. The Raman spectra of the samples were recorded in solid or powder using a Renishaw Raman RM2000 instrument equipped with an electrically cooled charge Coupled Device (CCD) camera and an excitation line using a 632.8 nm He/Ne laser.

Res and RLTA were placed in phosphate buffer solution (pH = 7.4) and artificial gastric juice (pH = 2.0), respectively, and the sustained release performance of RLTA was evaluated according to the mass reduction; these solutions mimic gastric acid and humoral conditions, respectively. Neither case involves proteases. RLTA and an equal amount of Res were added to one package. The package was immersed in a 500 mL solution and stirred at a 100 RPM paddle speed. The temperature was maintained at 37 °C. Each 1 mL sample was extracted at specific intervals (1 min, 5 min, 15 min, 30 min, 1 h, 2 h, 3 h, 4 h, 5 h, 6 h, 7 h, and 24 h) and the culture medium was supplemented with 1 mL diluted ChP or PBS after each sample. The concentration of resveratrol was determined by UV spectrophotometer at 308 nm. Three tests were conducted under the same conditions, and the data were shown as mean ± standard deviation. A release curve was drawn based on the mean and standard deviation of the three values at each time.

The drug loading efficiency was calculated by thermogravimetric analysis (TG) curve. TG-DTA curves were measured by synchronous thermal analyzer. Choosing air as the heating environment, the temperature drops 10 per minute. According to the mass loss at different temperatures, the endothermic and exothermic peaks at different temperatures, the drug loading efficiency can be estimated by Formula (1).

(1) $$\mathrm{Drug}\;\mathrm{Loading}\;(w/w)=\frac{\mathrm{total}\;\mathrm{weight}\;\mathrm{loss}-\mathrm{material}\;\mathrm{weight}\;\mathrm{loss}}{1-\mathrm{material}\;\mathrm{weight}\;\mathrm{loss}}\text{×}100\%$$

### 4.4. Animal Experiments

Twenty-five SD rats (male) aged 7 weeks were used in the experiment, which were purchased from Weitonglihua Experimental Animal Technology Co., LTD., and raised by Experimental Animal Center of Tongji University. Padding was replaced 2–3 times a week. The feeding temperature was 20–23 °C and the relative humidity was 50–60%. After feeding for one week, the rats were randomly divided into 5 groups with 5 rats in each group. The rats were weighed and recorded, and anesthetized with 10% chloral hydrate at a dose of 3 mL/kg. After anesthesia, the rats were injected with insulin needle at 0.1 mL/kg on the 1st, 4th and 7th day, followed by treadmill exercise for 3 weeks to aggravate inflammation. The first week of platform exercise is mainly for adaptation, and the amount of exercise is 10 m/min and 15 min. In the second week, exercise was performed at 13 m/min, 14 m/min and 15 m/min incrementally for 30 min. Modeling was completed after 3 weeks. After modeling, the rats were randomly divided into four groups: Cellulose group (Cellulose), Res group, Resveratrol—Cellulose aerogel delivery system group (C+R) and inflammatory group (OA), and Control group (Control). Control group was without intervention, while the OA group was given normal saline. The Cellulose group was given 8% Cellulose, the Res group was given 25 mg Res, the C+R group was given RLTA (the content of Res was 25 mg). Gavage wass carried out at a fixed time every day.

### 4.5. Biochemical Indexes

The removed fresh blood was added to the anticoagulant tube, removed with a 100 uL pipette gun, and placed into the reagent tray. The pipette gun was removed to avoid backpumping after all injections. The blood biochemical analyzer was turned on and the rat-adult-whole blood indexes were selected for detection. The test was finished 12 min later.

### 4.6. HE Staining

The sections were washed three times with PBS and fixed with 10% buffered formalin at 4 °C for 30 h. The slices were washed with distilled water for 5 min and washed under running water for 15 min, then stained with hematoxylin-eosin. Finally, the samples were cleaned with distilled water and examined under a confocal laser light microscope.

### 4.7. Micro-Computed Tomography (Micro-CT)

The rats to be tested were fixed in the Micro-CT examination slot and the scanning was started. The conditions were as follows: voltage 60 KV, current 666 pA. Images of different sections were obtained, and 3D image reconstruction was performed on the scan images of tibia and bone. After obtaining 3D in vivo imaging, the medial femoral condyle and medial tibial condyle of the knee of rats were selected in the image software to mark the distance between the two points, and the distance between the lateral femoral condyle and the lateral tibial condyle was also marked and recorded. The average distance between the medial and lateral condyles was calculated to obtain the length of the knee space, in nm. The difference of left and right joint space in the same rat and the difference of mean joint space among different groups of rats were analyzed and compared.

### 4.8. Enzyme-Linked Immunosorbent Assay (ELISA)

ELISA is used to measure the expression level of serum immune factors. The main method is to adsorb known antigens or antibodies on the surface of the solid phase carrier, and then incubate them with enzyme-labeled (coupled) antibodies or antigens, adding chromogenic agents to develop color. The difference between the color of the object to be measured and the color of the standard object was determined by the enzyme reader, and then the enzyme activity curve was drawn to obtain the concentration of the object to be measured.

### 4.9. Western Blot Analysis

Tissues were clipped and rinsed with ice-cold PBS and harvested in protein cracking liquid lysis buffer (Thermo Fisher Scientific) to obtain total cellular protein. Equal quantities of proteins were subjected to 5% SDS-PAGE (Thermo Fisher Scientific) and transferred to PVDF membranes (Sigma-Aldrich Corporation). Then, the membranes were blocked with 5% skim milk (0.1% TBST buffer) for 1 h at room temperature and were incubated with primary antibodies overnight at 4 °C. After that, the membranes were washed with TBST for three times, the secondary antibody was diluted 7000 times with TBST and incubated with blots for 60 min at room temperature. Finally, the Western blots were developed using ECL reagent through a Western blotting detection system.

### 4.10. Statistical Analysis

The measurement data in this experiment were recorded and analyzed using Microsoft Excel, and the data in each group were expressed as Mean ± Standard deviation (Mean ± SD). Origin 9.4 was used for the preparation of statistical charts and statistical analysis. Independent sample *t*-test was used for comparison between groups, and *p* < 0.05 indicated statistically significant differences.

## Figures and Tables

**Table 1:** Blood biochemical indexes of rats in each group.

	Cellulose	C+R	Res	OA	Control
ALB (g/L)	28.53 ± 0.90	29.20 ± 1.71	25.13 ± 1.30	25.90 ± 3.80	30.13 ± 1.40
TP (g/L)	63.07 ± 3.09	64.50 ± 4.71	61.43 ± 4.75	63.97 ± 3.94	63.07 ± 3.62
GLB (g/L)	36.20 ± 2.30	38.40 ± 7.93	40.53 ± 2.46	35.30 ± 0.10	33.90 ± 38.50
A/G	0.97 ± 0.07	0.76 ± 0.07	0.62 ± 0.01	0.73 ± 0.11	0.89 ± 0.91
STB(μmol/L)	2.10 ± 0.83	1.15 ± 0.15	1.05 ± 0.05	3.05 ± 2.05	2.07 ± 0.79
AMS(U/L)	513.00 ± 18.46	708.67 ± 39.47	909.67 ± 103.02	669.50 ± 184.50	772.00 ± 79.00
CK (U/L)	520.00 ± 167.48	1217.00 ± 906.00	780.00 ± 845.49	1173.50 ± 816.50	397.00 ± 38.00
TG (mmol/L)	0.69 ± 0.10	0.80 ± 0.18	0.54 ± 0.15	0.64 ± 0.34	0.61 ± 0.07
GLU (mmol/L)	9.53 ± 3.03	12.72 ± 3.95	11.57 ± 1.24	11.03 ± 0.40	13.46 ± 1.58
Ca (mmol/L)	0.61 ± 0.04	0.63 ± 0.01	0.63 ± 0.02	0.63 ± 0.04	0.64 ± 0.02
P (mmol/L)	2.51 ± 0.18	2.22 ± 0.02	2.31 ± 0.09	2.30 ± 0.09	2.35 ± 0.18
BUN/Cr	141.98 ± 16.50	195.92 ± 53.06	246.41 ± 31.68	199.10 ± 25.71	260.48 ± 22.38

## Data Availability

Data available on request from the authors.
